# Supplementary figures and images for: A minimal set of internal control genes for gene expression studies in head and neck squamous cell carcinoma
Source: PeerJ. 2018 Aug 14;6:e5207. doi: 10.7717/peerj.5207 (PMC6097490; doi:10.7717/peerj.5207)

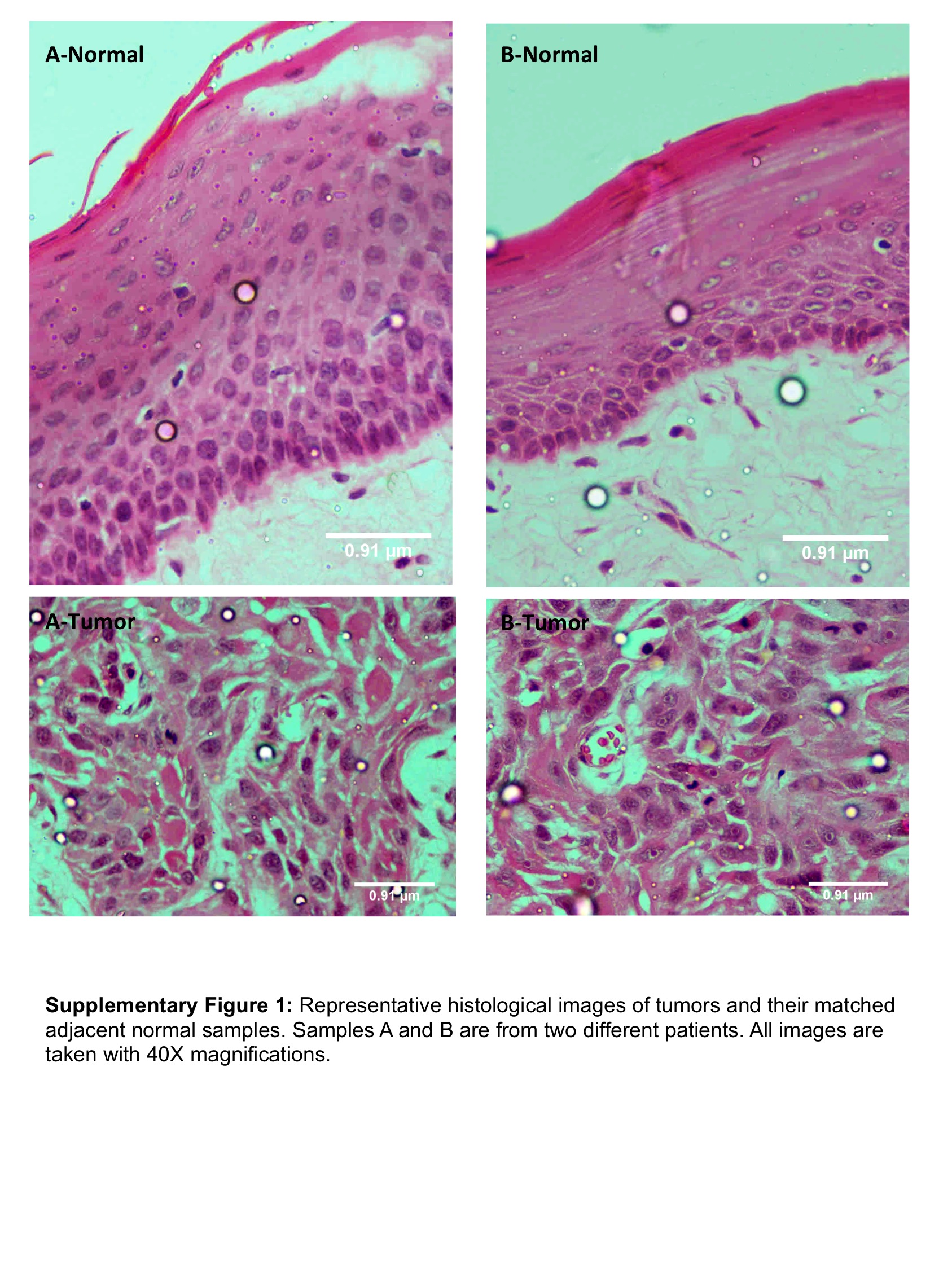

Supplement: Supplemental Information 1 [file peerj-06-5207-s001.jpg]

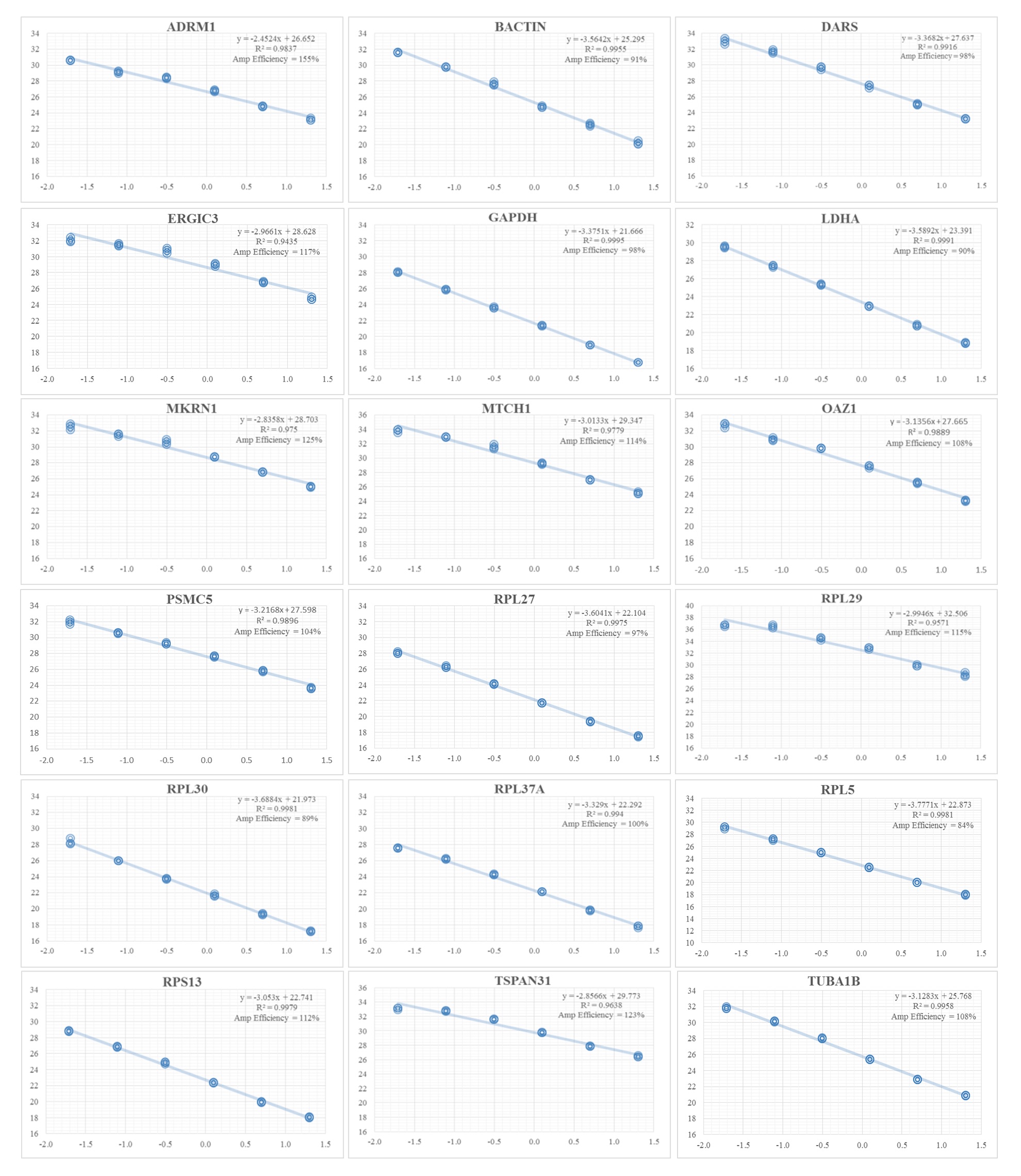

Supplement: Supplemental Information 2 — The qPCR was performed using 3 times dilution of the template. R2 indicates the correlation coefficient and amplification efficiencies on the basis of the slopes. [file peerj-06-5207-s002.jpg]

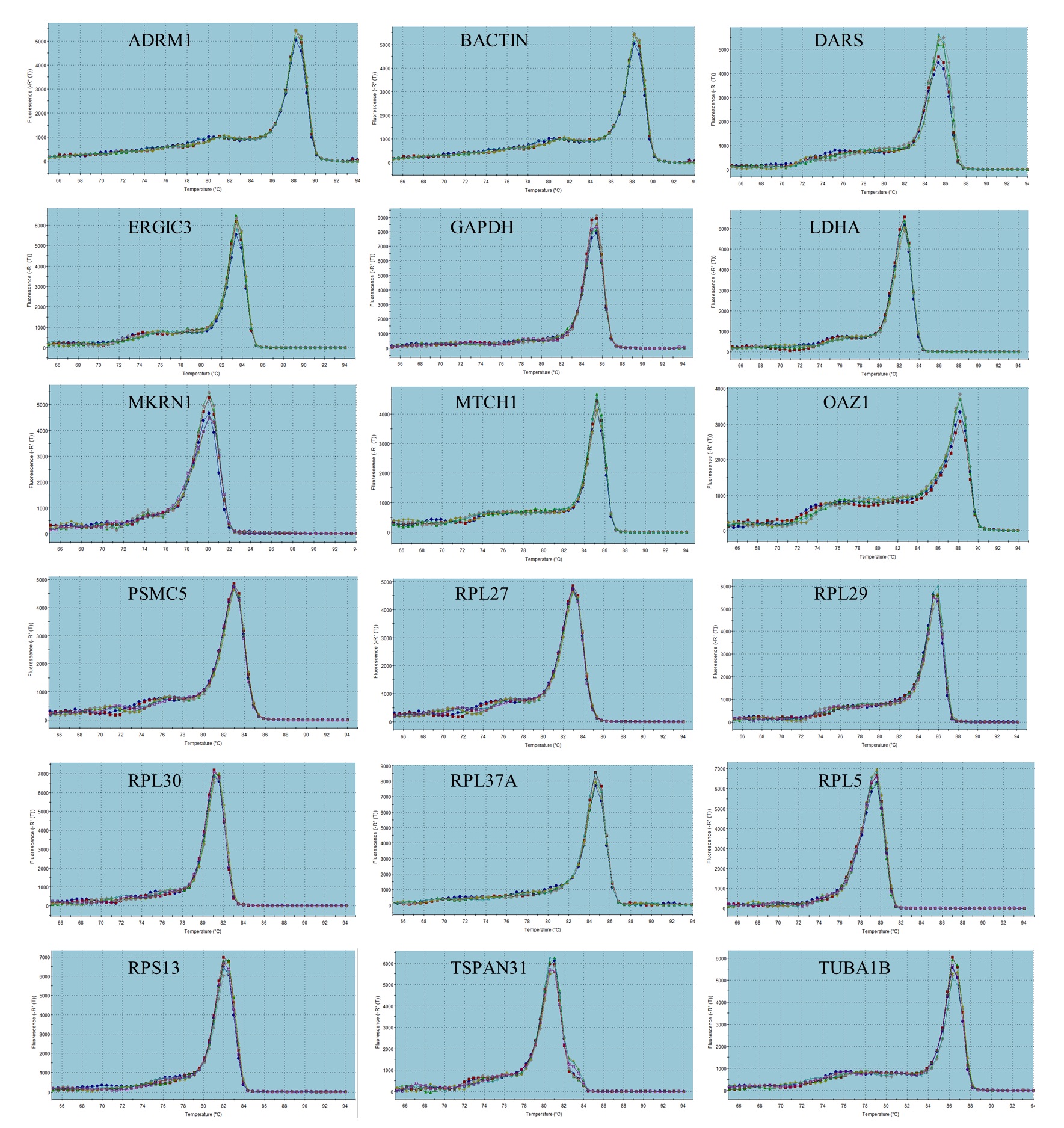

Supplement: Supplemental Information 3 — The single peak of dissociation curve in qPCR performed for all the genes indicates the specific amplification. [file peerj-06-5207-s003.jpg]

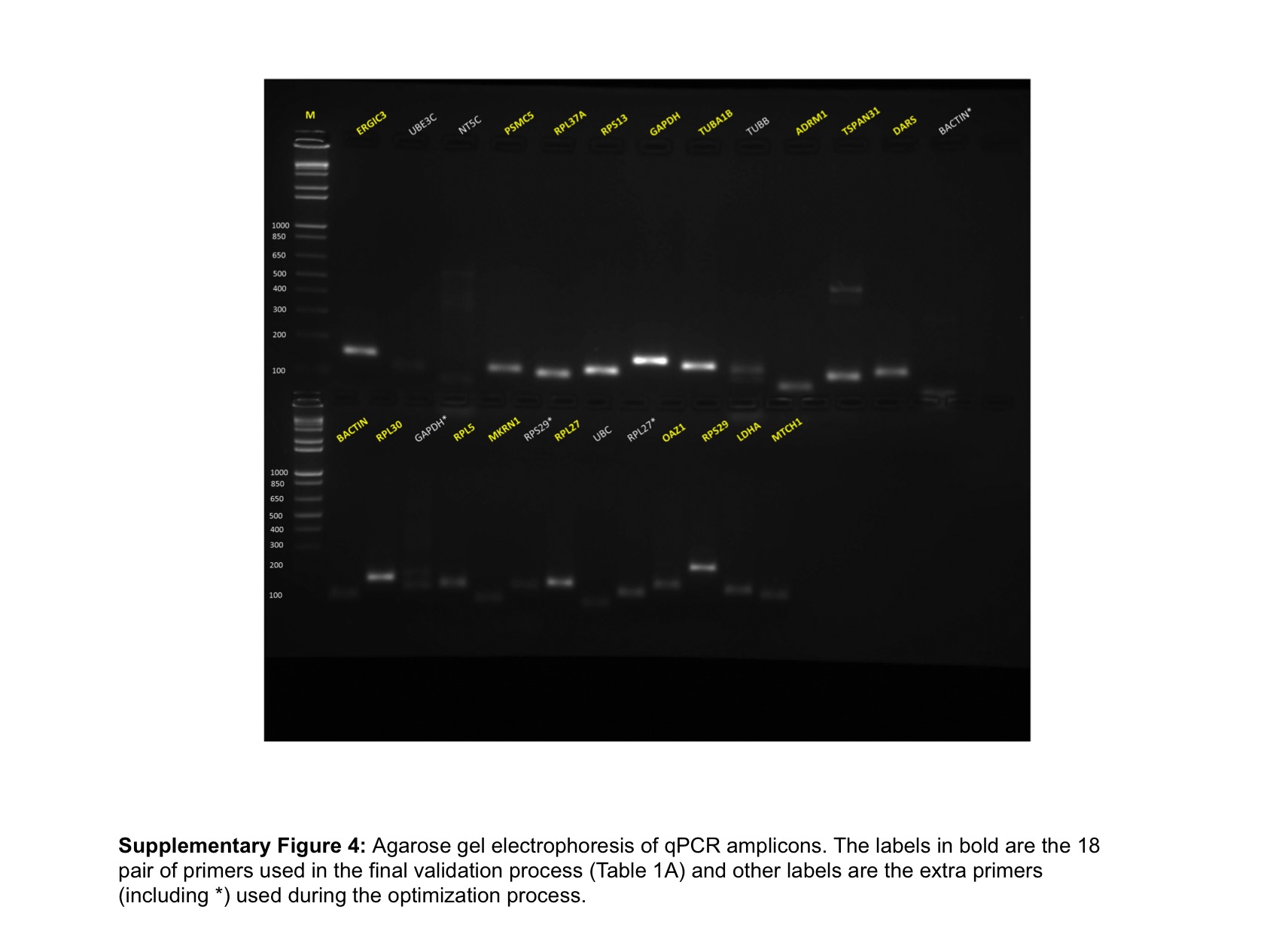

Supplement: Supplemental Information 4 — The labels in bold are the 18 pair of primers used in the final validation process (Table 1A) and other labels are the extra primers (including *) used during the optimization process. [file peerj-06-5207-s004.jpg]
